# Long‑term outcomes of adjustable gastric banding: a 15‑year prospective randomized trial comparing 2 band types in 103 patients 

**DOI:** 10.20452/wiitm.2024.17918

**Published:** 2024-12-11

**Authors:** Žygimantas Juodeikis, Gintautas Brimas

**Affiliations:** Clinic of Gastroenterology, Nephro-Urology and Surgery, Vilnius University, Vilnius, Lithuania

**Keywords:** adjustable gastric banding, bariatric and metabolic surgery, obesity

## Abstract

**INTRODUCTION:**

As the use of gastric bands diminishes in bariatric and metabolic surgery, we present the results of a 15-year randomized controlled trial comparing 2 distinct adjustable gastric bands.

**AIM:**

The aim of this study was to compare long-term outcomes of bariatric surgery performed using 2 different adjustable gastric band types over a 15-year period.

**MATERIALS AND METHODS:**

Between January 1, 2009, and January 31, 2010, a total of 103 patients with obesity underwent randomization to receive treatment with either a Swedish adjustable gastric band (SAGB; n = 49) or a MiniMizer Extra adjustable gastric band (n = 54). Weight loss outcomes, comorbidity resolution, long-term complications, and quality of life measures were assessed at 1, 5, and 15 years postoperatively.

**RESULTS:**

Baseline characteristics were similar between the groups, with a mean (SD) patient age of 45.9 (11.7) years and a mean (SD) preoperative body mass index of 47.5 (7.3) kg/m^2^ . Of the 103 patients, 55 (53.3%) completed the 15-year follow-up. After 15 years, the mean total body weight loss was 25.6% in the SAGB group and 20.6% in the MiniMizer Extra group, with no significant difference. Complications occurred in 19 patients (18.4%), including 5 band erosions, 4 port-related issues, 3 cases of band slippage, and 3 instances of band intolerance. Nine bands were removed, and 3 patients underwent conversion to gastric bypass.

**CONCLUSIONS:**

SAGB and MiniMizer Extra bands demonstrated comparable outcomes at both the 5- and 15-year follow-up with respect to weight loss, resolution of comorbidities, morbidity, and quality of life. However, most of the improvements in comorbidities observed at the 5-year follow-up significantly declined after 15 years.

## INTRODUCTION

Twenty years ago, the laparoscopic adjustable gastric banding procedure stood as a well-established and standardized restrictive method, prevailing as a primary bariatric and metabolic operation. However, its popularity waned, giving way predominantly to sleeve gastrectomy.^1^ Various adjustable gastric bands were utilized during this transition, each differing in design, filling volume, internal pressure, and fixation mechanism. Theoretical considerations suggest that these disparities might impact long-term outcomes. While numerous studies have compared different gastric band types, most focus on the Swedish adjustable gastric band (SAGB) and LAP-BAND devices, while the MiniMizer Extra adjustable band has received less attention.

The main difference between the SAGB and MiniMizer Extra bands lies in their fixation methods (with and without plication), which could potentially impact postoperative complication rates. This study serves as the first randomized controlled trial to compare these 2 devices, providing insights into their long-term effectiveness. Previous studies focused on patient outcomes at the 1- and 5-year follow-up, and did not reveal significant differences between these bands.^2^^;^^3^ Hence, we aimed to evaluate the extended outcomes of SAGB and MiniMizer Extra devices. Of note, data on the outcomes of bariatric and metabolic surgery beyond 15 years are rarely available in the medical literature.^4^ Therefore, our study is a valuable contribution to the long-term dataset.

## AIM

The aim of this study was to compare the long-term outcomes of bariatric surgery performed using 2 different adjustable gastric bands, SAGB and MiniMizer Extra, in morbidly obese patients over a 15-year period. The study focused on evaluating weight loss, resolution of comorbidities, long-term complications, and quality of life. By analyzing these outcomes, we intended to provide a clearer understanding of the long-term efficacy and safety of these 2 band systems in the context of bariatric surgery.

## MATERIALS AND METHODS

All individuals undergoing bariatric and metabolic surgery were invited to participate in the study. Between December 2009 and January 2010, a cohort of 103 patients with obesity underwent surgical procedures at the Center of Abdominal Surgery of the Vilnius University Hospital Santaros Klinikos in Vilnius, Lithuania.

The inclusion criteria encompassed age of 18 to 70 years and body mass index (BMI) exceeding 40 kg/m^2^ or BMI ranging from 35 to 40 kg/m^2^ accompanied by obesity-related comorbidities. The exclusion criteria comprised a history of prior bariatric and metabolic surgery, pregnancy, or other contraindications to laparoscopic procedures.

### Randomization process

The patients were randomly assigned in a 1:1 ratio to one of 2 parallel groups, receiving either the SAGB (Obtech Medical, Zürich, Switzerland) or the MiniMizer Extra (Bariatric Solutions GmbH, Münchenstein, Switzerland) band. Randomization involved patients choosing one of 2 identical and fully opaque envelopes containing the name of a band. The randomization process was double-blinded.

### Surgical technique and follow-up 

Laparoscopic gastric banding was performed using the pars flaccida technique. The SAGB was secured by creating a gastric fold over the band (plication), with 3 to 4 gastro-gastric 2–0 silk sutures. For the MiniMizer Extra band, the retaining loops were fixed directly to the anterior gastric wall using 5 interrupted 2–0 silk sutures (2 at the upper edge and 3 at the lower edge). The access port was implanted subcutaneously and anchored to the left rectus fascia using interrupted nonabsorbable sutures (MiniMizer Extra) or secured with a velocity device (SAGB). Both band systems were left unfilled at the time of surgery. In accordance with the study protocol, a comprehensive multidisciplinary evaluation was conducted 1, 5, and 15 years postoperatively. Band adjustments were not dictated by the study protocol but were instead based on individual weight loss outcomes.^2^^;^^3^

### Outcomes and measures

The primary end point of the study was weight loss, with secondary end points including complication rates, improvement in comorbidities, and quality of life. Preoperative evaluations were conducted by a multidisciplinary team comprising an endocrinologist, gastroenterologist, dietitian, cardiologist, and a bariatric and metabolic surgeon.

The evaluated comorbidities included diabetes mellitus (DM), arterial hypertension (AHT), cardiovascular disease (CVD), metabolic syndrome (MS), dyslipidemia, gastroesophageal reflux disease (GERD), and degenerative joint disease (DJD). Assessment of comorbidities relied on patient-reported information, clinical examination, and blood samples obtained after an overnight fast. Additionally, all patients underwent upper gastrointestinal endoscopy, abdominal ultrasonography, and upper gastrointestinal tract radiography. Criteria for resolution and improvement of comorbidities were adapted from the Bariatric Analysis and Reporting Outcome System (BAROS).^5^

DM was defined as a fasting plasma glucose level greater than or equal to 7.1 mmol/l, glycated hemoglobin A_1c_ concentration greater than or equal to 6.5%, or the use of antidiabetic medications.^6^ AHT was identified by a resting blood pressure exceeding 140/90 mm Hg or the use of antihypertensive therapy. CVD was diagnosed based on documented evidence of coronary artery disease, peripheral vascular disease, or congestive heart failure. Dyslipidemia was characterized by fasting low-density lipoprotein cholesterol concentrations greater than or equal to 3.3 mmol/l, high-density lipoprotein cholesterol levels below 1.03 mmol/l in men or below 1.29 mmol/l in women, triglyceride concentrations greater than or equal to 1.7 mmol/l, or the use of lipidlowering medications. MS was diagnosed in accordance with the Adult Treatment Panel III criteria.^7^ GERD was diagnosed based on upper gastrointestinal endoscopy findings, patient-reported symptoms, and the use of antireflux medications. DJD was identified through self-reported symptoms and previously documented evidence. 

Quality of life was evaluated using a modified version of the Moorehead-Ardelt Quality of Life II (M-AQoLII) questionnaire.^8^

### Sample size

Sample size calculation was conducted using the G*Power software (Heinrich-Heine Universität Düsseldorf, Düsseldorf, Germany). Based on prior assumptions, the mean (SD) percentage of excess weight loss (%EWL) at 5 years following gastric banding with the SAGB was estimated at 57% (1.9%), while for the MiniMizer Extra, it was estimated at 51% (15%). To detect a significant difference between the 2 groups, with a 2-tailed α of 0.05, a power of 80% (1-β), and an effect size of 0.56 for comparing 2 independent means, it was determined that a minimum of 102 participants would be required.^3^

**FIGURE 1:**
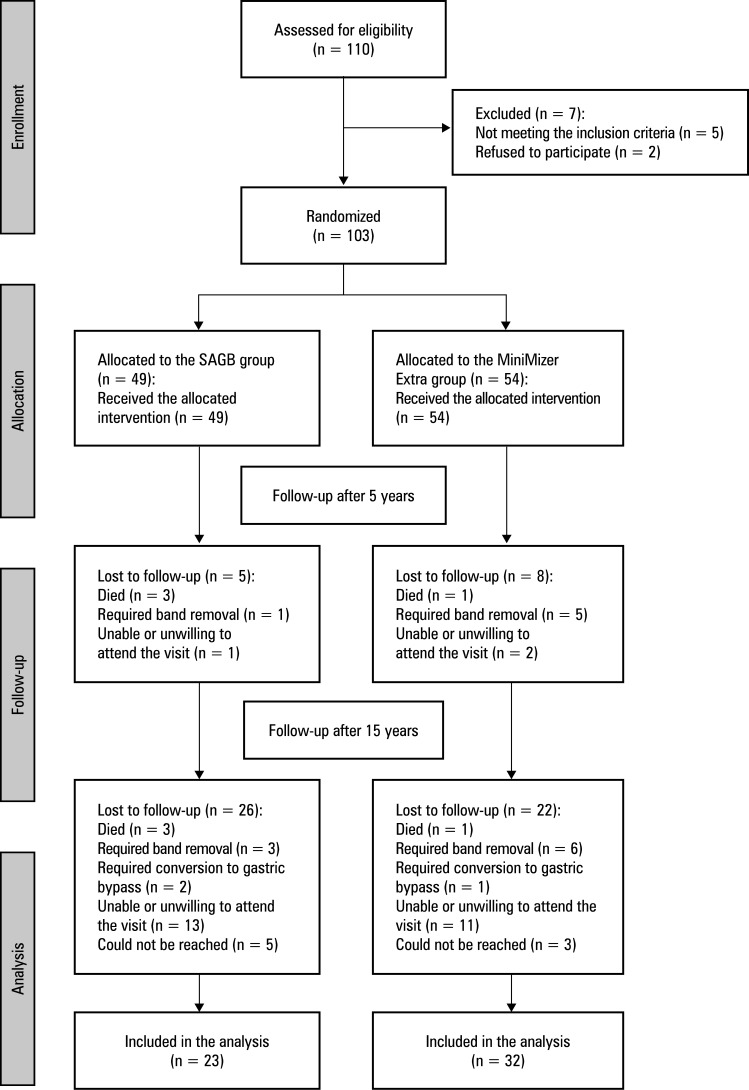
Study flow chart

### Statistical analysis

Statistical analysis was performed using SPSS package, version 21.0 (SPSS Inc., Chicago, Illinois, United States). The Pearson χ^2^ test or Fisher exact test was used to test for differences between categorical variables, and the 2-sample *t* test or Mann–Whitney test was used for comparison of continuous variables, depending on distribution. The Shapiro–Wilk test was used to check the normality of data distribution. A *P* value below 0.05 was considered significant.

### Ethics

The study protocol received approval from the Lithuanian Bioethics Committee (#L-08-62, protocol No. 1). All patients provided written informed consent to participate in the study.

The study was conducted in accordance with the ethical standards of the institutional and national research committees and with the 1964 Helsinki Declaration and its later amendments.

**TABLE 1:** Baseline patient characteristics

Parameter		All patients	SAGB	MiniMizer Extra	*P *value
Patients, n		103	49	54	0.62
Age, y		45.9 (11.7)	46.1 (11.5)	45.8 (11.9)	0.87
Sex, n (%)	Women	69 (67)	31 (63.6)	38 (70.4)	0.53
	Men	34 (33)	18 (36.7)	16 (29.6)	0.94
Body weight, kg		137.6 (24.4)	141.8 (24.2)	133.8 (24)	0.09
BMI, kg/m^2^		47.5 (7.3)	48.6 (7.9)	46.5 (6.7)	0.16
EBMI, kg/m^2^		22.5 (7.4)	23.6 (7.9)	21.5 (6.7)	0.17
EW, kg		64.9 (21.2)	68.5 (22.1)	61.8 (20)	0.11

Data are presented as mean (SD) unless indicated otherwise.

Abbreviations: BMI, body mass index; EBMI, excess BMI; EW, excess weight; others, see FIGURE 1

**TABLE 2:** Weight loss parameters 5 and 15 years postsurgery

Parameter	5-year follow-up (all patients)	15-year follow-up (all patients)	*P *value
Weight, kg	107.6 (26.2)	105.2 (23.6)	0.22
BMI, kg/m^2^	37.1 (8.3)	36.9 (8)	0.27
BMIL, kg/m^2^	10.1 (6.7)	11.2 (7.1)	0.27
TBWL, kg	28.8 (18.3)	32.0 (20.2)	0.22
%TBWL	21.2 (13.2)	23.1 (13.4)	0.29
%EWL	47.3 (29.7)	49.7 (28.5)	0.32

Data are presented as mean (SD).

Abbreviations: BMIL, body mass index loss; %EWL, percentage excess weight loss;

TBWL, total body weight loss; %TBWL, percentage total body weight loss; others, see TABLE 1

**TABLE 3:** Weight loss parameters 15 years postsurgery

Parameter	All patients	SAGB	MiniMizer Extra	*P *value
Weight, kg	105.2 (23.6)	103.3 (21.8)	107 (25.5)	0.59
BMI, kg/m^2^	36.9 (8)	36.2 (8.4)	37.4 (7.7)	0.6
BMIL, kg/m^2^	11.2 (7.1)	12.7 (7.8)	9.8 (6.3)	0.16
TBWL, kg	32 (20.2)	36.3 (21.4)	27.8 (18.5)	0.37
%TBWL	23.1 (13.4)	25.6 (14)	20.6 (12.7)	0.2
%EWL	49.7 (28.5)	54.9 (30.2)	44.8 (26.3)	0.22

Data are presented as mean (SD).

Abbreviations: see FIGURE 1 and TABLE 1  andTABLE 2

## RESULTS

The study flow chart is presented inFIGURE 1. A cohort of 103 patients (69 women [67%], 34 men [33%]) was randomly allocated to either the SAGB or the MiniMizer Extra group. The mean (SD) preoperative age of the whole cohort was 45.9 (11.7) years, and the mean (SD) preoperative BMI was 47.5 (7.3) kg/m^2^. Baseline characteristics of the 2 groups are outlined inTABLE 1 .

Of the initial 103 patients, 55 (53.3%) completed the 15-year follow-up. Four individuals (3.9%) died to unrelated causes, while 9 (8.7%) underwent band removal due to complications, and 3 (2.9%) required conversion to gastric by pass. The deaths were attributed to lung cancer, pancreatic cancer, and acute cardiovascular events occurring between 1 to 4 years postoperatively. The patients whose bands were removed or who underwent conversion to gastric bypass were ex cluded from the analyses of weight loss and comorbidity resolution. The remaining patients that were lost to follow-up either refused to continue participating in the trial, were unable to attend follow-up visits, or could not be reached.

On average, the patients underwent 6.16 band adjustments over 5 years and 9.1 adjustments over 15 years, with a trend toward more adjustments in the SAGB group, as compared with the MiniMizer Extra group (9.7 vs 6.5; *P* = 0.07). 

Weight loss parameters at the 5- and 15-year follow-up are outlined inTABLE 2 andTABLE 3. The mean (SD) percentage of total body weight loss (%TBWL) was 21.2% (13.2) after 5 years and 23.1% (13.4) after 15 years, with no significant difference between the 2 bands.

The baseline distribution of comorbidities is delineated inTABLE 4 . At the 5-year follow-up, improvements or resolution were observed with respect to DM (55.5% of the affected patients), AHT (53%), CVD (50%), dyslipidemia (78.5%), GERD (67.7%), and DJD (40.3%). Additionally, a resolution in metabolic syndrome was noted in 62.3% of the affected patients. However, a majority of the improvements substantially diminished after 15 years. There were no significant differences observed between the 2 bands concerning the resolution and improvement of comorbiditiesTABLE 5. 

At the 15-year follow-up, the overall complication rate was 18.4%, with no significant disparity observed between the MiniMizer Extra and SAGB groupsTABLE 6. Complications encompassed 5 instances of band erosions, 4 port-related issues, 3 occurrences of band slippage, 3 instances of band intolerance, and 4 cases of suboptimal weight loss. Port-related complications comprised 3 cases of port-site infections and 1 instance of port inversion, none of which were associated with band erosion. Surgical intervention was necessary for managing complications in 16 patients (15.5%). Nine bands were removed (3 cases in the SAGB group and 6 cases in the MiniMizer Extra group; *P* = 0.12), with 3 removals attributed to erosions (all in the MiniMizer Extra patients) and 6 to patient psychological intolerance and suboptimal weight loss (2 in the SAGB group and 4 in the MiniMizer Extra group). All 3 instances of band slippage were managed by laparoscopic band repositioning. Furthermore, all instances of port infections necessitated the removal and subsequent reimplantation of the port. 

**TABLE 4:** Comorbidities at the baseline

Parameter	Total (n = 103)	SAGB (n = 49)	MiniMizer Extra (n = 54)	*P *value
DM	33 (32)	19 (38.7)	14 (25.9)	0.16
AHT	82 (79.6)	35 (71.4)	47 (87)	0.05
CVD	21 (20.3)	11 (22.4)	10 (18.5)	0.62
Dyslipidemia	69 (66.9)	36 (73.4)	33 (62.2)	0.22
MS	69 (66.9)	33 (67.3)	36 (66.6)	0.63
GERD	45 (43.6)	21 (42.8)	24 (44.4)	0.87
DJD	71 (68.9)	35 (71.4)	36 (66.6)	0.6

Data are resented as number (percentage).

Abbreviations: AHT, arterial hypertension; CVD, cardiovascular disease; DJD, degenerative joint disease; DM, diabetes mellitus; GERD, gastroesophageal reflux disease; MS, metabolic syndrome; others, seeFIGURE 1

**TABLE 5:** Resolution and improvement of comorbidities 5 and 15 years postsurgery

Parameter	Resolution /improvement at the 5-year follow-up			Resolution /improvement at the 15-year follow-up		
	SAGB	MiniMizer Extra	*P *value	SAGB	MiniMizer Extra	*P *value
DM	10 (62.4)	5 (35.6)	0.53	5 (35.1)	3 (25.3)	0.49
AHT	16 (49.9)	19 (41.2)	0.72	9 (49.9)	12 (32.3)	0.81
CVD	3 (33.3)	4 (40)	0.28	2 (22.2)	2 (20)	0.3
Dyslipidemia	25 (86.2)	19 (70.3)	0.14	17 (68.9)	10 (47.3)	0.12
MS	20 (60.6)	23 (63.8)	0.79	12 (54.7)	14 (52.8)	0.33
GERD	13 (68.3)	11 (33.3)	0.37	8 (63.4)	6 (31.1)	0.27
DJD	8 (25.9)	16 (45.7)	0.81	4 (12.4)	8 (37.6)	0.62

Data are presented as number (percentage).

Abbreviations: see FIGURE 1 andTABLE 4

**TABLE 6:** Complications at the 15-year follow-up

Adverse event	Total	SAGB	MiniMizer Extra	*P *value
Band erosion	5 (4.8)	0	5 (9.2)	0.06
Band slippage	3 (2.9)	2 (4)	1 (1.8)	0.61
Band intolerance	3 (2.9)	1 (2)	2 (3.7)	0.51
Port-related issues	4 (3.8)	1 (2)	3 (5.5)	0.36
Suboptimal weight loss	4 (3.8)	2 (4)	2 (3.7)	0.36
Total	19 (18.4)	6 (12)	13 (24)	0.08

Data are presented as number (percentage).

Abbreviations: seeTABLE 1

The mean (SD) M-AQoLII score significantly improved from 0.02 (1.2) at the baseline to 1 (1.2) after 5 years (*P* <0.001). This trend in enhanced quality of life continued after 15 years, with mean (SD) scores reaching 1.3 (1.2) (*P* = 0.11). Notably, no significant differences were observed between the SAGB and MiniMizer extra groups.

The mean (SD) BAROS score after 5 years was 2.92 (2.5) points in the SAGB group and 3.22 (2.1) points in the MiniMizer Extra group (*P* = 0.47), indicating favorable outcomes. The average score remained similar after 15 years, with 2.95 (2.4) points in the SAGB group and 3.25 (2) points in the MiniMizer Extra group (*P* = 0.45).

## DISCUSSION

The ascent of gastric banding was rapid, matched only by its subsequent decline. The inaugural laparoscopic adjustable gastric band was introduced in 1993, quickly gaining popularity worldwide due to its simplicity and a favorable safety profile.^9^ By 2003, adjustable gastric banding accounted for approximately 24.4% of all bariatric procedures, rising to around 42.3% by 2008, second only to gastric bypass.^10^ However, the emergence of sleeve gastrectomy as an independent bariatric procedure, offering superior weight loss outcomes and lower complication rates, contributed to the gradual decline of gastric banding. Consequently, by 2013, gastric banding comprised only about 10% of all bariatric surgeries, plummeting further to just 0.8% by 2022.^1^

This randomized controlled trial comparing the outcomes of 2 distinct bands was initiated in 2009, a period when gastric banding was at the peak of its popularity. There was a specific interest in comparing various aspects of band designs and assessing whether differences in band design, filling volume, internal pressure, and fixation mechanism impact the outcomes.

The reported occurrence of late complications in high-volume centers ranged from 6% to 25%.^11^ The most frequently documented long-term complications included band slippages, band erosions, and port-related issues. In our study, the incidence of late complications was 18.4%. The reported incidence of band erosion in a systematic review by Egberts et al^12^ was 1.4%. In our study, the incidence of band erosion was higher, at 4.8%. Interestingly, all 5 cases of band erosion occurred in the MiniMizer Extra group, suggesting that the differences in band design might contribute to a higher rate of this complication. However, the difference between the bands was not significant (9.2% vs 0%; *P* = 0.06). All 5 cases of band erosion were observed during the initial follow-up period (1–5 years postsurgery), with no new erosions developing after 15 years.

In our study, 3 patients (2.9%) experienced band slippage at an average of 55 months post-surgery. No significant difference was observed between the SAGB and MiniMizer Extra groups with respect to this complication (4% vs 1.8%; *P* = 0.6). All 3 instances of band slippage were successfully managed through laparoscopic band repositioning, with an uncomplicated postoperative course.

The average %EWL after 15 years in both groups was 49.7%, with no significant difference between the groups. Interestingly, our results are similar to those obtained by O’Brien et al,^4^ who reported a %EWL of 47% after 15 years.^4^

As anticipated, a majority of improvements in comorbidities observed at the 5-year follow-up gradually decreased after 15 years, with no notable differences between the bandsTABLE 5.

Surprisingly, the average quality of life score at 15 years was better than the 5-year results. However, this finding must be tempered by the fact that only 53% of the patients remained in the analysis at the 15-year follow-up. There is a strong possibility that patients who fail to attend follow-up visits have lower quality of life.

The findings of this study have significant implications for the current landscape of bariatric and metabolic surgery. As adjustable gastric banding has largely been replaced by more effective and durable procedures, such as sleeve gastrectomy and gastric bypass,^1^ our results provide critical insight into the long-term viability and limitations of gastric banding.

The similar weight loss achieved with both types of bands, along with the relatively high rate of complications, emphasizes the limitations of gastric banding as a durable treatment option for morbid obesity. Moreover, the improvements in comorbidities seen during the first 5 years after the operation gradually decreased, pointing to the limited effectiveness of gastric banding in the long term. These findings contribute to the ongoing discussion about the role of gastric banding in contemporary bariatric and metabolic surgery. They show the importance of careful patient selection and quality protocols of follow-up when considering gastric banding as a treatment option while highlighting the necessity of prioritizing procedures that provide greater durability and improved long-term outcomes.

This study has several limitations. First, it was performed in a single center, which may impact the generalizability of the results. Second, band adjustments and postoperative care were individualized rather than standardized, which may have impacted long-term outcomes. Lastly, advancements in bariatric surgery and weight management over the past 15 years could limit the applicability of these results to current clinical practice. Despite these limitations, our study adds valuable insight to the expanding body of research that can help shape future approaches to obesity treatment and ensure that patients receive the best care possible.

## CONCLUSIONS

The SAGB and MiniMizer Extra bands demonstrated comparable outcomes at both the 5- and 15-year follow-up with respect to the resolution of comorbidities, morbidity, and quality of life. Weight loss was also similar between the 2 groups. However, a majority of improvements in comorbidities observed at the 5-year follow-up notably declined after 15 years.
